# Dynamic Assessment of the C-Reactive Protein-to-Albumin Ratio Improves Early Prediction of Severe Acute Pancreatitis: A Retrospective Cohort Study

**DOI:** 10.3390/life16091564

**Published:** 2026-09-17

**Authors:** Crina Fofiu, Laura Nat, Andrei Constantin Ioanovici, Alina Boeriu

**Affiliations:** 1 Gastroenterology Department, George Emil Palade University of Medicine, Pharmacy, Science, and Technology of Târgu Mureș, 540142 Targu Mures, Romania; alina.boeriu@umfst.ro; 2 Internal Medicine Department, Bistrita County Clinical Hospital, 420094 Bistrita, Romania; 3 Complementary Functional Sciences, Medical Informatics and Biostatistics, George Emil Palade University of Medicine, Pharmacy, Science, and Technology of Târgu Mureș, 540142 Târgu Mureș, Romania; andrei.ioanovici@umfst.ro

**Keywords:** acute pancreatitis, C-reactive protein-to-albumin ratio, serial biomarkers, severity prediction, risk stratification

## Abstract

**Background/Objectives**: Previous studies have demonstrated the prognostic value of the C-reactive protein-to-albumin ratio (CAR) in acute pancreatitis, but the incremental value of serial measurements remains unclear. We evaluated whether CAR measured at 24 and 48 h provides additional prognostic information beyond admission CAR for predicting severe acute pancreatitis (SAP). **Methods**: In this retrospective cohort study, 150 consecutive patients with acute pancreatitis were included. CAR was calculated at admission (CAR-T0), 24 h (CAR-T24), and 48 h (CAR-T48). Univariable and multivariable logistic regression, receiver operating characteristic (ROC) analysis, and DeLong’s test were used to evaluate prognostic performance. Secondary analyses explored associations with pancreatic pseudocyst, pancreatic ascites, and splanchnic venous thrombosis. **Results**: Forty-four patients (29.3%) developed SAP. CAR was significantly associated with SAP at all three time points, with CAR-T24 demonstrating the highest predictive performance (AUC 0.874), followed by CAR-T48 (AUC 0.857) and CAR-T0 (AUC 0.829). CAR-T24 showed the highest individual discriminative performance, although its AUC was not significantly higher than that of CAR-T0 (DeLong *p* = 0.067). In contrast, adding CAR-T24 to the prespecified multivariable model significantly improved discrimination compared with the admission model (AUC 0.890 vs. 0.841; *p* = 0.016). Dynamic changes in CAR (ΔCAR) were not independently associated with SAP. Admission NLR showed poor predictive performance (AUC 0.578) and was significantly inferior to all CAR time points. Exploratory analyses suggested a potential association between CAR-T24 and splanchnic venous thrombosis; however, given the small number of thrombotic events (*n* = 10), this finding should be considered preliminary and hypothesis-generating. **Conclusions**: Serial CAR assessment, particularly at 24 h after admission, provides incremental prognostic value beyond admission CAR alone and may provide additional prognostic information during early clinical reassessment in acute pancreatitis.

## 1. Introduction

Acute pancreatitis (AP) is one of the leading causes of gastrointestinal emergency hospital admissions worldwide, characterized by a highly variable clinical course ranging from mild, self-limiting disease to severe acute pancreatitis complicated by persistent organ failure, infected necrosis, and substantial mortality. Although approximately 80% of patients experience a favorable outcome, up to one-fifth develop moderately severe or severe disease, associated with prolonged hospitalization, intensive care unit admission, local complications, and increased healthcare costs. Consequently, early and accurate risk stratification is critical to guide appropriate management [1,2,3].

Several prognostic scoring systems for acute pancreatitis severity have been developed, including the Ranson score, APACHE II, Bedside Index of Severity in Acute Pancreatitis (BISAP), and modified Computed Tomography Severity Index (MCTSI) [4,5,6].

However, their clinical utility is constrained by complexity, requirement for multiple clinical and laboratory parameters, need for 48 h data completion, or dependence on CT availability, limiting their routine use in emergency settings [2,3,7,8]. Consequently, interest has grown in simple, rapid, and cost-effective biomarkers measurable at any point during the disease course for early risk stratification. The C-reactive protein-to-albumin ratio (CAR) and neutrophil-to-lymphocyte ratio (NLR) are both derived from routine admission blood tests, reflecting the intensity of the inflammatory response and the host’s nutritional and immunological status, respectively [9,10,11,12]. Their early availability and low cost make them attractive candidates for bedside severity assessment.

Elevated CRP values reflect the acute inflammatory stimulus. Previous research has shown that both admission and 48 h CRP demonstrate limited value in predicting complicated acute pancreatitis [9]. Albumin is a negative acute-phase reactant whose hepatic synthesis is suppressed in inflammatory states. Hypoalbuminemia has been correlated with increased disease severity, organ failure, and mortality in AP [13,14].

By combining these two complementary markers, the C-reactive protein-to-albumin ratio (CAR) provides a more comprehensive reflection of the inflammatory and nutritional status. Its prognostic value in sepsis, critically ill patients, and AP has been demonstrated across multiple studies, with higher CAR values associated with SAP, pancreatic necrosis, organ failure, prolonged hospitalization, and mortality [15,16,17,18]. Many of these reports have focused on a single CAR measurement at admission [19,20,21].

However, acute pancreatitis is a dynamic inflammatory disease characterized by rapid changes in inflammatory activity during the first 48–72 h after symptom onset. Serial CAR assessment may therefore provide additional prognostic information beyond baseline values, capturing the trajectory of the inflammatory response. We hypothesized that CAR kinetics over the first 24–48 h of hospitalization would better reflect the evolving inflammatory response and improve early prediction of severe acute pancreatitis and its complications.

The neutrophil-to-lymphocyte ratio (NLR) emerged as a prognostic tool in oncology and critical care before its role in acute pancreatitis was investigated [22,23]. Elevated NLR in SAP reflects the combined effects of neutrophilia driven by bone marrow stimulation from SIRS-associated cytokines and lymphopenia resulting from both cortisol-mediated lymphocyte apoptosis and redistribution to inflamed tissues. Several studies and meta-analyses have evaluated NLR as a predictor of AP severity, ICU admission, and in-hospital mortality [24,25,26].

However, few studies have examined the prognostic value of admission NLR and serial CAR within the same patient cohort, or directly compared their discriminative performance across the clinical spectrum of AP complications.

Previous studies have established the prognostic value of CAR in acute pancreatitis, and more recent evidence has also demonstrated associations between CAR measured at different time points during the early disease course and adverse outcomes [27]. However, whether reassessment of CAR provides statistically demonstrable incremental prognostic information beyond the admission value within the same prediction model remains insufficiently characterized. Furthermore, the prognostic relevance of the absolute CAR value at standardized reassessment time points compared with the arithmetic change from baseline (ΔCAR) has received limited investigation.

Accordingly, the novelty of the present study does not reside in the serial measurement of CAR per se, but in evaluating whether serial CAR reassessment adds prognostic information beyond admission CAR. We therefore assessed the incremental contribution of CAR-T24 and CAR-T48 to a baseline multivariable model containing admission CAR and established clinical predictors, formally compared model discrimination, and evaluated whether absolute serial CAR values or their changes from baseline provide greater prognostic information.

Therefore, the primary aim of this study was to determine whether CAR reassessment at 24 and 48 h provides incremental prognostic value for severe acute pancreatitis beyond admission CAR and established baseline predictors. Secondary objectives were to compare the prognostic performance of absolute serial CAR values with changes from baseline (ΔCAR), to compare CAR with admission NLR, and to explore associations with selected complications.

## 2. Materials and Methods

### 2.1. Study Design and Patient Selection

This retrospective cohort study was conducted at Bistrița County Emergency Hospital, Romania, and included consecutive adult patients admitted with acute pancreatitis (AP) between 1 January and 31 December 2024. The diagnosis of AP was established according to the 2012 Revised Atlanta Classification [1] and required the presence of at least two of the following three criteria: (1) characteristic abdominal pain consistent with AP; (2) serum lipase and/or amylase concentrations at least three times the upper limit of normal; and (3) imaging findings compatible with AP on abdominal ultrasonography or contrast-enhanced computed tomography (CECT). Patients were excluded if they had a prior diagnosis of chronic pancreatitis, active malignancy (including pancreatic carcinoma), severe chronic liver disease (Child-Pugh B or C cirrhosis), chronic immunosuppressive therapy, or other conditions considered likely to substantially interfere with the interpretation of inflammatory biomarkers. A total of 150 patients fulfilled the eligibility criteria and were included in the final analysis.

The study cohort included 40 patients (26.7% of the present 150-patient cohort) whose admission data had been included in a previously published preliminary analysis [28]. These patients were admitted between January and August 2024 and are therefore a subset of the current cohort. The previous publication evaluated CAR only at the initial assessment and did not report serial CAR measurements or analyses of the incremental prognostic value of CAR reassessment.

The study was approved by the Ethics Committee of Bistrița County Emergency Hospital (approval number: 12674/4, dated 13 August 2025) and conducted in accordance with the Declaration of Helsinki. Informed consent was waived given the retrospective, non-interventional nature of the study.

### 2.2. Clinical and Laboratory Data Collection

Clinical and laboratory data were extracted from electronic medical records using a standardized, predefined data collection form. Demographic variables included age and sex. Clinical characteristics comprised the mode of presentation (emergency admission vs. inter-hospital transfer), symptoms at presentation (epigastric pain, nausea, vomiting, and paralytic ileus), etiology of AP (biliary, alcohol-related, autoimmune, or drug-induced), and length of hospital stay.

Routine laboratory parameters were collected at admission and during hospitalization. Variables included serum C-reactive protein (CRP), serum albumin, complete blood count with differential (including absolute neutrophil and lymphocyte counts), serum lipase, blood urea nitrogen (BUN), serum creatinine, alanine aminotransferase (ALT), and aspartate aminotransferase (AST). CRP and serum albumin were available for all 150 patients at three predefined time points: hospital admission (T0), 24 h after admission (T24), and 48 h after admission (T48).

### 2.3. Calculation of Inflammatory Biomarkers

The C-reactive protein-to-albumin ratio (CAR) was calculated by dividing serum CRP concentration (mg/L) by serum albumin concentration (g/L). Albumin concentrations recorded in the source dataset in g/dL were converted to g/L by multiplying by 10 prior to all calculations. CAR was calculated at three predefined time points: hospital admission (CAR-T0), 24 h after admission (CAR-T24), and 48 h after admission (CAR-T48). To quantify the dynamic trajectory of CAR, two delta variables were defined: ΔCAR-T24 = CAR-T24 − CAR-T0, representing the change from admission to 24 h; and ΔCAR-T48 = CAR-T48 − CAR-T0, representing the change from admission to 48 h. Positive delta values indicate a rising inflammatory burden relative to admission; negative values indicate attenuation of the inflammatory response. The neutrophil-to-lymphocyte ratio (NLR) was calculated as the absolute neutrophil count divided by the absolute lymphocyte count at admission.

### 2.4. Radiological Assessment

All 150 patients underwent contrast-enhanced computed tomography (CECT) during hospitalization, performed at least 72 h after symptom onset when clinically indicated, to allow optimal delineation of pancreatic necrosis. Imaging findings were extracted from existing radiological reports; dedicated re-review by a study radiologist was not performed, which represents a potential limitation. Pancreatic morphology was classified as interstitial edematous or necrotizing pancreatitis according to the 2012 Revised Atlanta Classification [1]. In necrotizing pancreatitis, the extent of necrosis was recorded as less than 30%, 30–50%, or greater than 50% of the pancreatic parenchyma. Radiological severity was assessed using the Modified Computed Tomography Severity Index (MCTSI), which incorporates the degree of pancreatic inflammation, the extent of pancreatic necrosis, and the presence of extrapancreatic complications, with higher scores indicating greater radiological severity [29].

### 2.5. Assessment of Acute Pancreatitis Severity and Clinical Outcomes

Disease severity was classified according to the 2012 Revised Atlanta Classification. Severe acute pancreatitis (SAP) was defined by persistent organ failure lasting more than 48 h in at least one of the respiratory, cardiovascular, or renal systems. Organ failure was assessed using the modified Marshall scoring system and was considered present when a score of ≥2 was recorded for any of these organ systems. Mild and moderately severe acute pancreatitis were combined as the non-severe comparison group for the purposes of the present analyses.

Secondary clinical outcomes were predefined and extracted retrospectively from the medical records and imaging reports. In-hospital mortality was defined as death from any cause occurring during the index hospitalization. Intensive care unit (ICU) admission was recorded when transfer to the ICU occurred during the index hospitalization. Length of hospital stay was calculated from the date of hospital admission to discharge or in-hospital death. Nasogastric decompression was recorded when nasogastric tube placement was required because of clinically documented paralytic ileus.

Local and vascular complications were identified from contrast-enhanced computed tomography (CECT) reports obtained during the index hospitalization. Pancreatic pseudocyst was recorded when a documented encapsulated fluid collection consistent with a pseudocyst was present on imaging. Splanchnic venous thrombosis was defined as documented thrombosis involving the portal, splenic, and/or mesenteric veins. Pancreatic ascites was recorded when ascites attributed to the acute pancreatic process was documented on CECT. Analyses involving these individual complications were considered exploratory because of their relatively low event frequencies.

### 2.6. Statistical Analysis

Continuous variables were summarized as median and interquartile range (IQR), and categorical variables as counts and percentages. Disease severity was classified according to the 2012 Revised Atlanta Classification. Severe acute pancreatitis (SAP) was defined by persistent organ failure lasting more than 48 h in at least one of the respiratory, cardiovascular, or renal systems. Organ failure was assessed using the modified Marshall scoring system and was considered present when a score of at least 2 was recorded for any of these organ systems. Mild and moderately severe acute pancreatitis were combined as the non-severe comparison group.

The C-reactive protein/albumin ratio (CAR) was calculated as CRP (mg/L) divided by albumin (g/L). Because albumin values in the source dataset were recorded in g/dL, they were multiplied by 10 before calculation and were reported in g/L.

Univariable binomial logistic regression was used to evaluate the association between SAP and CAR at admission (CAR-T0), 24 h (CAR-T24), and 48 h (CAR-T48), as well as the absolute changes from admission to 24 h (ΔCAR-T24 = CAR-T24 − CAR-T0) and from admission to 48 h (ΔCAR-T48 = CAR-T48 − CAR-T0). Discrimination was assessed using the area under the receiver operating characteristic curve (AUC).

Three multivariable logistic regression models were prespecified and fitted without data-driven variable selection. The admission model included age, blood urea nitrogen (BUN), and CAR-T0. The primary serial model added CAR-T24 to the admission model and represents prognostic reassessment after 24 h. A secondary model added CAR-T48 instead and was interpreted as a later reassessment rather than an admission triage model. All continuous predictors were retained on their original continuous scales. The incremental contribution of CAR-T24 and CAR-T48 was assessed using likelihood-ratio comparisons with the admission model. Odds ratios (ORs) with 95% confidence intervals (CIs) were reported, and collinearity was assessed using variance inflation factors. The locked-model variables were complete for all 150 patients; therefore, no imputation was performed.

Internal validation was performed using enhanced bootstrap optimism correction with 2000 resamples and a prespecified random seed (20260819). In each bootstrap sample, the complete locked model was refitted, and its performance was evaluated both in that bootstrap sample and in the original cohort. The mean difference represented optimism and was subtracted from the apparent performance. We report apparent and optimism-corrected AUC, Brier score, calibration intercept, and calibration slope. Apparent AUC 95% CIs were obtained by percentile bootstrap resampling of patient pairs. Calibration was additionally displayed in ten equally sized groups with Wilson 95% CIs for the observed event proportion. A bootstrap-derived uniform shrinkage factor was estimated for the primary 24 h model. Random split-sample validation was not used because it would inefficiently divide a cohort of 150 patients with 44 SAP events.

Analyses were performed in jamovi version 2.6.45 using binomial logistic regression and the Rj module with R version 4.4.1. All tests were two-sided, with *p* < 0.05 considered statistically significant. No independent cohort was available; consequently, external validation was not performed.

## 3. Results

### 3.1. Baseline Characteristics of the Study Population

A total of 150 patients were included, of whom 44 (29.3%) met criteria for severe acute pancreatitis and 106 (70.7%) had non-severe disease. Baseline demographic, clinical, and laboratory characteristics are summarized in Table 1.

Patients with SAP were more frequently male (72.7% vs. 50%, *p* = 0.017), although no significant difference in age was observed between groups (median 57 vs. 53 years, *p* = 0.176). Biliary pancreatitis was the most common etiology (56.0%), followed by alcohol-related pancreatitis (37.3%), with autoimmune, drug-induced, and other causes accounting for the remainder.

Regarding clinical outcomes, ICU admission was required in 20 patients (13.3%), and in-hospital mortality occurred in 3 patients (2.0%), both significantly more frequent in the SAP group (*p* < 0.001 and *p* = 0.0024, respectively). Among local complications, pancreatic ascites was the most frequent, occurring in 45 patients (30.0%), followed by pancreatic pseudocyst in 12 (8.0%) and splanchnic venous thrombosis in 10 (6.7%). Patients with SAP had a significantly higher prevalence of splanchnic venous thrombosis compared with those with non-severe disease (18.2% vs. 1.9%, *p* = 0.001). No statistically significant between-group differences were observed for pancreatic pseudocyst (13.6% vs. 5.7%, *p* = 0.111) or pancreatic ascites (34.1% vs. 28.3%, *p* = 0.558).

Patients who developed SAP exhibited higher inflammatory marker levels and lower albumin concentrations throughout the first 48 h of hospitalization (Table 1). Median CRP was significantly higher in the SAP group at admission [228.5 (IQR 159.5–271.0) vs. 126.5 (IQR 62.75–167.0) mg/L, *p* < 0.001], at 24 h [210.0 (IQR 172.25–258.75) vs. 120.0 (IQR 65.0–153.5) mg/L, *p* < 0.001], and at 48 h [123.0 (IQR 82.0–156.0) vs. 40.5 (IQR 23.0–75.0) mg/L, *p* < 0.001]. Serum albumin was consistently lower in SAP patients at all three time points [T0: 33.5 vs. 36.0 g/L; T24: 29.0 vs. 34.0 g/L; T48: 27.0 vs. 32.0 g/L; all *p* < 0.001]. Accordingly, CAR was markedly higher in the SAP group at T0 [6.87 (IQR 4.63–8.66) vs. 3.48 (IQR 1.61–4.60), *p* < 0.001], T24 [7.32 (IQR 5.31–8.81) vs. 3.33 (IQR 1.81–4.64), *p* < 0.001], and T48 [4.28 (IQR 2.92–5.75) vs. 1.25 (IQR 0.68–2.39), *p* < 0.001]. MCTSI scores were also significantly higher in the SAP group [median 8 (IQR 8–9) vs. 4 (IQR 4–5), *p* < 0.001].

### 3.2. Prognostic Performance of Serial CAR for Severe Acute Pancreatitis

**Figure 1:**
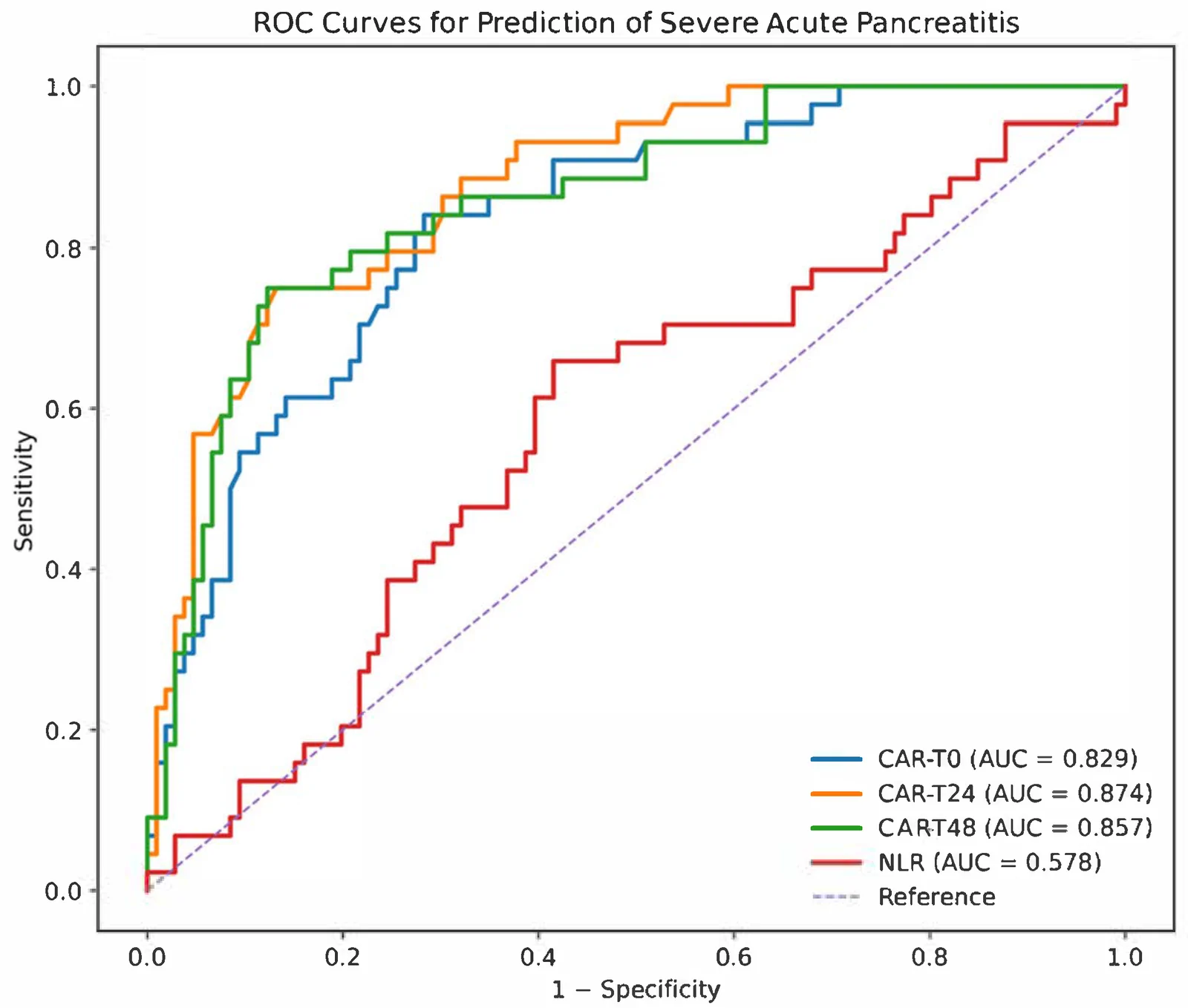
ROC curves for prediction of SAP.

In univariable logistic regression analysis, CAR at all three time points was significantly associated with SAP. Admission CAR (CAR-T0) yielded an OR of 1.663 (95% CI 1.384–1.998; *p* < 0.001) and an AUC of 0.829 (95% CI 0.761–0.898). CAR at 24 h (CAR-T24) demonstrated the highest discriminative performance among the evaluated time points, with an OR of 1.926 (95% CI 1.554–2.386; *p* < 0.001) and an AUC of 0.874 (95% CI 0.816–0.932), though the difference in AUC relative to CAR-T0 did not reach statistical significance (DeLong’s test: *p* = 0.067). CAR at 48 h (CAR-T48) was similarly associated with SAP (OR 2.121, 95% CI 1.645–2.735; *p* < 0.001; AUC 0.857, 95% CI 0.791–0.922) (Figure 1). Optimal cutoff values, sensitivity, and specificity for each time point are presented in Table 2.

In contrast, neither ΔCAR-T24 (OR 1.196, 95% CI 0.937–1.526; *p* = 0.151) nor ΔCAR-T48 (OR 0.838, 95% CI 0.683–1.027; *p* = 0.089) was significantly associated with SAP. The OR below 1.0 for ΔCAR-T48 suggests that patients whose CAR decreased between admission and 48 h were less likely to develop SAP, consistent with attenuation of the inflammatory response in milder disease; however, this association did not reach statistical significance.

In multivariable analysis, the reference model comprising age, BUN, and CAR-T0 achieved an AUC of 0.841 (95% CI 0.773–0.909). In this model, BUN (OR 1.05, 95% CI 1.00–1.10; *p* = 0.040) and CAR-T0 (OR 1.65, 95% CI 1.36–2.00; *p* < 0.001) were independently associated with SAP, whereas age did not reach statistical significance (*p* = 0.636) (Table 3).

Addition of CAR-T24 to the reference model significantly improved model fit (likelihood ratio test: *p* < 0.001) and increased the AUC to 0.890 (95% CI 0.834–0.945; DeLong’s test vs. reference model: *p* = 0.016). In this extended model, CAR-T24 was independently associated with SAP (OR 1.92, 95% CI 1.39–2.65; *p* < 0.001). CAR-T0 was no longer independently significant (*p* = 0.864), a finding consistent with the moderate collinearity between admission and 24 h CAR values (VIF = 3.61).

Similarly, addition of CAR-T48 to the reference model significantly improved model fit (likelihood ratio test *p* = 0.002) and increased the AUC to 0.868 (95% CI 0.806–0.931; DeLong’s test vs. reference model: *p* = 0.130). CAR-T48 remained independently associated with SAP (OR 1.65, 95% CI 1.18–2.31; *p* = 0.004), whereas neither CAR-T0 nor BUN retained statistical significance in this model.

Overall, the addition of serial CAR measurements, particularly CAR-T24, which yielded the highest AUC of 0.890, provided incremental prognostic discrimination beyond admission CAR alone. ΔCAR variables did not contribute additional predictive value in this cohort.

### 3.3. Association of Serial CAR Measurements with Local Complications

The associations of serial CAR and NLR measurements with local complications, including pancreatic pseudocyst, pancreatic ascites, and splanchnic venous thrombosis, were evaluated in exploratory logistic regression and ROC analyses (Table 4).

Given the limited number of events for individual complications, all findings in this section should be considered exploratory and hypothesis-generating rather than as evidence of established predictive performance.

Neither CAR-T24 nor CAR-T48 was significantly associated with the development of pancreatic pseudocyst. CAR-T24 yielded an OR of 1.14 (95% CI 0.93–1.40; *p* = 0.211) and an AUC of 0.587 (95% CI: 0.409–0.764), and CAR-T48 similarly showed no significant predictive value (OR 1.15, 95% CI 0.89–1.48; *p* = 0.284; AUC 0.585, 95% CI: 0.400–0.770).

No significant association was observed between serial CAR and pancreatic ascites: AUC values were 0.505 and 0.519 for CAR-T24 and CAR-T48, respectively.

In contrast, both CAR-T24 and CAR-T48 were significantly associated with splanchnic venous thrombosis in univariable analyses. CAR-T24 yielded an OR of 1.69 (95% CI 1.28–2.24; *p* < 0.001) and an AUC of 0.825 (95% CI 0.651–0.999); CAR-T48 demonstrated comparable association (OR 1.65, 95% CI 1.24–2.18; *p* < 0.001; AUC 0.819, 95% CI 0.688–0.949). However, these results must be interpreted cautiously given that thrombosis occurred in only 10 patients (6.7%), resulting in wide confidence intervals and limited statistical power.

In summary, serial CAR measurements were not significantly associated with pancreatic pseudocyst or pancreatic ascites. The observed association between elevated serial CAR and splanchnic venous thrombosis is an exploratory finding that warrants prospective validation in larger cohorts.

### 3.4. Comparative Performance of CAR and NLR

The median NLR at admission (NLR-T0) in the overall cohort was 5.16 (IQR 2.52–9.57). Patients with SAP demonstrated higher NLR values than those with non-severe disease [median 6.13 (IQR 3.26–10.00) vs. 4.59 (IQR 2.44–7.93)], although this difference did not reach statistical significance (*p* = 0.131).

ROC analysis demonstrated poor discriminative performance of admission NLR for identifying SAP, with an AUC of 0.578 (95% CI 0.477–0.680). The optimal Youden threshold was NLR ≥ 5.29, corresponding to a sensitivity of 65.9% and a specificity of 58.5%. Direct comparison of ROC curves demonstrated that both admission CAR (CAR-T0) and 24 h CAR (CAR-T24) significantly outperformed NLR for predicting SAP (CAR-T0: AUC 0.829 vs. 0.578, ΔAUC 0.251, 95% CI 0.127–0.375, *p* < 0.001; CAR-T24: AUC 0.874 vs. 0.578, ΔAUC 0.296, 95% CI 0.173–0.419, *p* < 0.001), favoring CAR as the superior prognostic biomarker for SAP in this cohort (Table 5).

**Figure 2:**
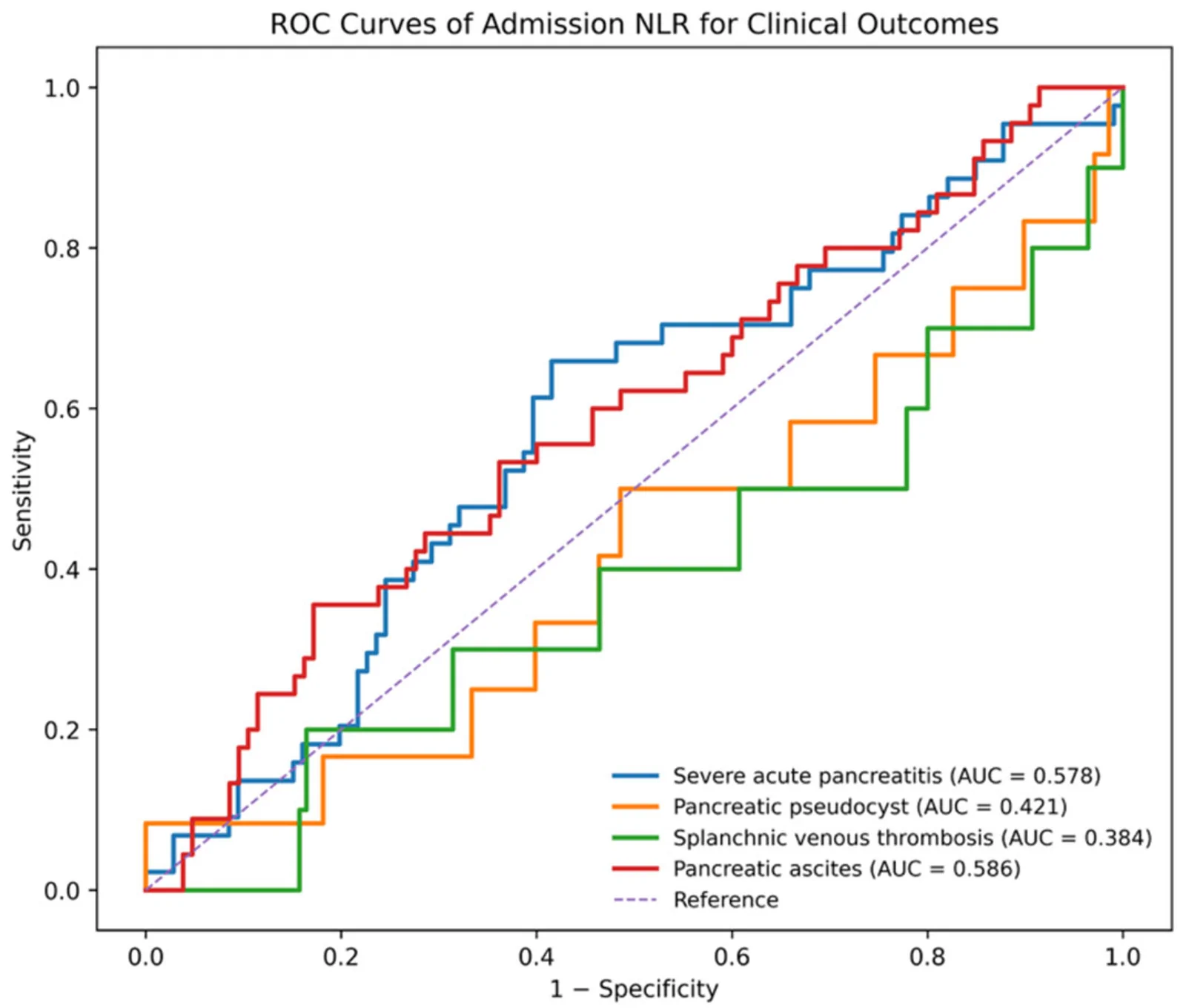
ROC curves for NLR as a predictor of local complications.

NLR was also evaluated as a predictor of local complications; no significant associations were identified for pancreatic pseudocyst (OR 1.10, 95% CI 0.95–1.27; *p* = 0.210), splanchnic venous thrombosis (OR 0.74, 95% CI 0.39–1.40; *p* = 0.351), or pancreatic ascites (OR 1.02, 95% CI 0.90–1.16; *p* = 0.714). AUC values for individual complications ranged from 0.38 for splanchnic vein thrombosis to 0.59 for pancreatic ascites, a finding that reflects the small event numbers and the exploratory nature of these analyses (Figure 2).

### 3.5. Internal Validation of the Multivariable Models

All 2000 bootstrap repetitions were successfully completed for each locked model (Table 6). The coefficients and equations of the locked logistic regression models are provided in Table S1. The optimism-corrected AUC was 0.831 for the admission model, 0.877 for the primary 24 h model, and 0.853 for the secondary 48 h model, compared with apparent AUCs of 0.841, 0.890, and 0.868, respectively. The corresponding optimism-corrected Brier scores were 0.147, 0.127, and 0.136. Corrected calibration intercepts were close to zero (0.005, 0.013, and 0.008), while corrected calibration slopes were 0.953, 0.919, and 0.920, indicating modest overfitting. Thus, the 24 h serial model retained the best discrimination and overall accuracy after internal validation.

Serial CAR measurement, particularly at 24 h, added prognostic information beyond age, BUN, and admission CAR in this cohort. Delta CAR did not outperform the raw serial measurements. These findings represent internal validation only; clinical transportability and decision thresholds require assessment in an independent cohort before the model can be used for routine triage or management decisions.

Internal validation used 2000 enhanced bootstrap repetitions in 150 patients, including 44 severe acute pancreatitis events. The complete prespecified model was refitted in every bootstrap sample. Apparent AUC confidence intervals are percentile bootstrap intervals. A calibration intercept of 0 and slope of 1 indicate ideal calibration; a lower Brier score indicates better overall probabilistic accuracy. Abbreviations: AUC, area under the receiver operating characteristic curve; BUN, blood urea nitrogen; CAR, C-reactive protein/albumin ratio; CI, confidence interval.

**Figure 3:**
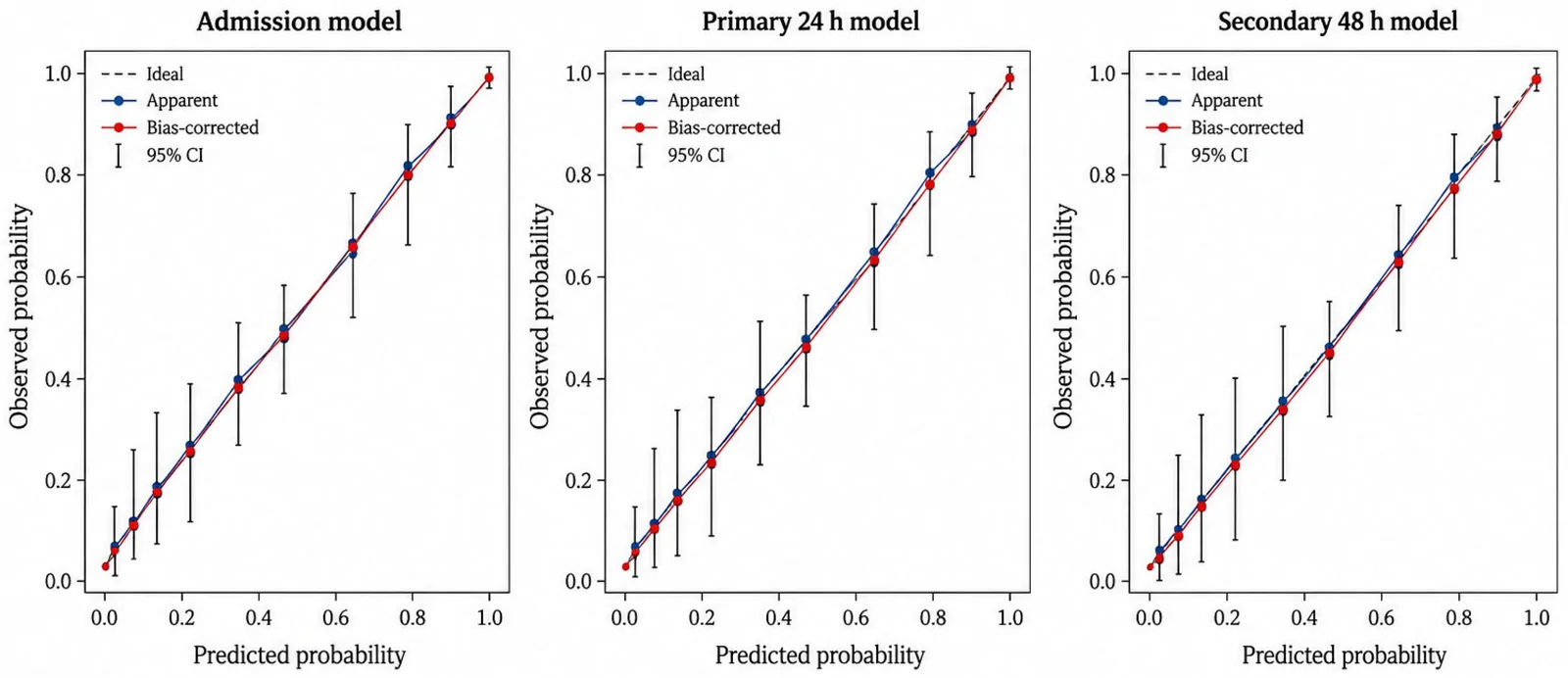
Calibration of the admission, primary 24 h, and secondary 48 h models after internal bootstrap validation.

The dashed line indicates ideal calibration (Figure 3); the optimism-corrected logistic calibration curve is shown for each model. Points show observed event proportions in ten equally sized groups with Wilson 95% confidence intervals.

## 4. Discussion

The present study demonstrates that serial assessment of the C-reactive protein-to-albumin ratio (CAR) during the first 48 h of hospitalization provides clinically meaningful prognostic information for the early identification of patients at risk of severe acute pancreatitis. Among the serial measurements evaluated, CAR at 24 h after admission demonstrated the strongest discriminative performance (AUC 0.874) and remained independently associated with SAP after adjustment for age, BUN, and admission CAR in multivariable analysis. However, CAR-T24 should be interpreted as a dynamic reassessment marker rather than an admission triage marker, as initial treatment and early clinical evaluation have already occurred by this time point. Although CAR-T24 yielded the highest individual AUC among the evaluated CAR time points, its univariable discriminative performance was not statistically superior to that of CAR-T0 (AUC 0.874 vs. 0.829; DeLong *p* = 0.067). Importantly, however, incorporation of CAR-T24 into the prespecified multivariable model including age, BUN, and CAR-T0 significantly improved discrimination (AUC 0.890 vs. 0.841; *p* = 0.016), supporting its incremental prognostic contribution in the multivariable setting rather than a statistically demonstrated superiority as a standalone marker. This finding is biologically consistent with the known kinetics of CRP, which typically peaks 48 h after the onset of systemic inflammation [30], suggesting that admission CRP, and consequently admission CAR, may not fully reflect the evolving inflammatory response at the time of presentation. Although admission CAR was independently associated with SAP in the reference model, its independent contribution was attenuated after inclusion of CAR-T24. Of note, dynamic changes in CAR (ΔCAR) were not independently associated with SAP, suggesting that the absolute level of inflammatory burden at 24 h is more prognostically informative than the rate of change from admission; this may be partly attributable to inter-individual variability in baseline CRP levels. By contrast, admission NLR demonstrated poor discriminative performance for SAP in this cohort (AUC 0.578).

Importantly, the prognostic performance of the prespecified multivariable models was further assessed by internal validation using enhanced bootstrap optimism correction. The relatively small decrease in discrimination after optimism correction suggests limited model optimism, particularly for the primary 24 h model, whose AUC decreased from 0.890 to 0.877. This model also retained the lowest optimism-corrected Brier score (0.127), while its corrected calibration intercept (0.013) remained close to zero and the calibration slope (0.919) indicated only modest overfitting. Taken together, these findings support the internal robustness of the 24 h reassessment model and strengthen the observation that CAR-T24 provides prognostic information beyond age, BUN, and admission CAR. Nevertheless, internal validation cannot establish model transportability across different clinical settings or patient populations.

Our findings extend the existing evidence supporting the prognostic utility of CAR in AP by demonstrating the incremental value of serial over single-point assessment. Unlike CRP or albumin assessed in isolation, CAR integrates two complementary dimensions of the acute systemic response. CRP reflects innate immune activation, with its hepatic synthesis driven by interleukin-6 and other pro-inflammatory cytokines. Albumin, conversely, decreases through a combination of cytokine-mediated suppression of hepatic synthesis, increased vascular permeability with protein redistribution to extravascular compartments, and nutritional depletion. CAR therefore simultaneously captures both the intensity of the inflammatory response and the host’s physiological reserve, offering a more comprehensive assessment of disease severity [15,16,17,18,31].

Several studies have previously demonstrated the prognostic value of admission CAR in AP. Kaplan et al. first reported that CAR > 16, 28 predicted mortality risk with 92.1% sensitivity and 58% specificity [15]. Behera et al. subsequently confirmed that elevated admission CAR was independently associated with persistent organ failure, pancreatic necrosis, prolonged hospitalization, and severe disease per the Revised Atlanta Classification [18]. Further retrospective studies from multiple world regions have produced consistent findings, reporting that CAR demonstrates better discriminatory performance than CRP alone for identifying patients at risk of severe dise [18,20,21,27,32]. Notably, however, none of these studies evaluated the prognostic contribution of serial CAR reassessment beyond the admission time point, nor did they formally compare CAR with NLR within the same cohort.

Our findings confirm that admission CAR is an independent predictor of severe acute pancreatitis, consistent with prior evidence. However, the present study extends this knowledge by demonstrating that the prognostic performance of CAR is not static but evolves during the early inflammatory phase of the disease. In the multivariable analysis, incorporation of CAR-T24 increased the AUC from 0.841 to 0.890 (DeLong’s test: *p* = 0.016), and CAR-T24 emerged as the strongest independent predictor in the extended model. The attenuation of admission CAR in this model reflects the moderate collinearity between repeated measurements rather than a true loss of independent predictive value, as indicated by the VIF analysis. This finding has a clear biological basis. Patients present at variable intervals after symptom onset, introducing substantial inter-individual variability that limits the discriminative power of a single admission value. Reassessment at 24 h post-admission represents a more standardized time point, by which cytokine release, IL-6-driven acute-phase protein synthesis, endothelial dysfunction, and increased vascular permeability are more uniformly manifest across patients [1,2,9,10,33,34,35,36]. Consequently, CAR at this time point may more reliably reflect the true inflammatory burden and its physiological consequences.

Our results reinforce a broader principle emerging in critical illness research: serial laboratory reassessment provides prognostic information that single baseline measurements cannot capture. This has previously been demonstrated for blood urea nitrogen [8], CRP [10], and composite inflammatory indices in critically ill patients [16,17,36]. Zhao et al. have recently studied dynamic changes in CAR for the prediction of SAP and death, demonstrating that CAR at 24 h was associated with SAP and pancreatic necrosis but was not a useful predictor of organ failure and mortality. CAR at 48 h was associated with SAP (OR 1.74, 95% CI: 1.32 to 2.29) death (OR: 1.73, 95% CI: 1.24 to 2.41), pancreatic necrosis (OR: 1.28, 95% CI: 1.08 to 1.50), and organ failure (OR: 1.43, 95% CI: 1.18 to 1.730), while CAR on day 3 was associated with a higher risk of SAP (OR: 1.50, 95% CI: 1.24 to 1.81), death (OR: 1.8, 95% CI: 1.34 to 2.65), pancreatic necrosis (OR: 1.22, 95% CI: 1.04 to 1.42), and organ failure (OR: 1.21, 95% CI: 1.04 to 1.4 [27].

We demonstrated that the absolute CAR value at 24 h outperformed the dynamic change in CAR (ΔCAR) as a predictor of SAP. This observation merits pathophysiological consideration. During the early inflammatory phase of AP, the systemic response is driven by acinar cell injury, DAMP release, and macrophage and neutrophil activation, leading to a steep rise in pro-inflammatory cytokines, particularly IL-1β, IL-6, and TNF-α. IL-6 stimulates hepatic synthesis of CRP, while albumin decreases due to reduced hepatic synthesis, increased vascular permeability, and redistribution. Consequently, CAR reflects both the intensity of inflammation and the physiological consequences of systemic illness [31,34,35,37]. In patients destined to develop SAP, both the CRP rise and the albumin fall are more pronounced and sustained. At 24 h, the absolute CAR therefore already reflects this divergence in inflammatory magnitude. The delta measurement, by contrast, captures only the increment from an admission value that itself varies substantially depending on the time from symptom onset to presentation. Patients admitted early in the disease course will show a larger ΔCAR simply due to the natural kinetic rise in CRP, regardless of disease severity.

As noted above, the superior performance of CAR-T24 over admission CAR is consistent with the kinetics of CRP and albumin during acute systemic inflammation: CRP peaks at approximately 48 h, while albumin declines more gradually, such that the 24 h measurement better reflects the established inflammatory state than an admission value taken at a variable and often early phase of the response [9,10,35]. The temporal kinetics argument therefore applies equally to explaining both the superiority of CAR-T24 over CAR-T0 and also the limited value of ΔCAR.

The absence of an independent association between ΔCAR and SAP deserves specific consideration, as this is, to our knowledge, the first study to formally evaluate dynamic CAR changes as a prognostic variable in AP. Several post-hoc hypotheses may account for this observation. First, the absolute CAR value at 24 h may already integrate both baseline inflammatory burden and early disease evolution, rendering the level reached at this time point more informative than the increment from admission. Second, an arithmetic difference between two measurements is inherently dependent on the starting value and may amplify biological and analytical variability: patients with markedly different admission CAR values may exhibit similar absolute changes while remaining at very different levels of inflammatory burden. Third, albumin concentration is influenced by factors beyond inflammation, including baseline nutritional status, hepatic synthetic reserve, vascular permeability, and therapeutic interventions. Intravenous fluid resuscitation, in particular, induces intravascular volume expansion and hemodilution, which may substantially alter measured albumin concentrations independently of true inflammatory activity. These factors may attenuate the prognostic signal of ΔCAR relative to the absolute time-point value. Accordingly, our negative ΔCAR findings do not argue against serial biomarker assessment; rather, they suggest that the absolute CAR value at a clinically relevant reassessment time point is more informative than the arithmetic change from baseline.

In contrast to CAR, NLR demonstrated poor prognostic discrimination in the present cohort. Although NLR values were numerically higher in patients with SAP (median 6.13 vs. 4.59), this difference did not reach statistical significance (*p* = 0.131), and the AUC for prediction of SAP was 0.578 (95% CI 0.477–0.680). Previous studies have reported heterogeneous findings regarding the prognostic value of NLR in AP, with variation in patient selection, timing of blood sampling, severity classification systems, and proposed threshold values likely contributing to inconsistent results across cohorts [24,25,26,38,39].

The difference in discriminative performance between CAR and admission NLR was substantial across all time points evaluated. Both CAR-T0 (AUC 0.829) and CAR-T24 (AUC 0.874) significantly outperformed admission NLR (AUC 0.578), with differences confirmed using DeLong’s method (*p* < 0.001 for both comparisons). This superiority may reflect the broader biological information encoded by a ratio combining a positive acute-phase reactant (CRP) with a negative acute-phase reactant (albumin), compared with a leukocyte subset-derived index that captures only one dimension of the inflammatory response. An important limitation of the present comparison, however, is that CAR was assessed serially across three time points whereas NLR was evaluated only at admission. Several cohort-specific factors should also be acknowledged, as they may have independently influenced NLR values regardless of disease severity: the high proportion of biliary AP (56%), the effects of early intravenous fluid resuscitation on leukocyte counts, and potential early antibiotic exposure. A direct prospective comparative evaluation incorporating serial measurements of both CAR and NLR within the same cohort is therefore recommended before definitive conclusions can be drawn regarding the prognostic utility of NLR in SAP. Bengi et al. demonstrated that NLR retained prognostic value when assessed at 24 and 48 h, predicting SAP with 79% sensitivity and 67% specificity at 24 h, and correlating with mortality and organ failure at 48 h [24].

An additional finding of the present study was the association between serial CAR measurements and splanchnic venous thrombosis. In exploratory analyses, both CAR-T24 and CAR-T48 were significantly associated with thrombotic complications (AUC 0.825 and 0.819, respectively), whereas CAR was not significantly associated with pancreatic pseudocyst or pancreatic ascites (AUC 0.505–0.587). A relationship between systemic inflammation and splanchnic thrombosis is biologically plausible. Acute pancreatitis may promote thrombosis through local inflammatory extension, endothelial injury, venous compression, platelet activation, hypercoagulability, and neutrophil-mediated thrombo-inflammatory mechanisms, including neutrophil extracellular trap (NET) formation [40,41,42,43,44]. Higher CAR values may therefore identify patients with a more pronounced systemic inflammatory and prothrombotic phenotype. In contrast, pseudocyst formation and pancreatic ascites are predominantly influenced by local anatomical factors, the extent and location of pancreatic injury, ductal disruption, and the evolution of peripancreatic fluid collections, which may be less directly captured by an inflammatory biomarker.

These thrombosis findings must, however, be interpreted with considerable caution. Only 10 thrombotic events occurred in the present cohort (6.7%), and the wide confidence intervals around both AUC and OR estimates reflect this limited statistical power. The association between serial CAR and splanchnic venous thrombosis should therefore be considered hypothesis-generating rather than evidence supporting a complication-specific prediction model or changes in imaging surveillance practice. If replicated in larger, multicenter cohorts with adequate event numbers, this observation may justify systematic investigation of CAR as one component of future strategies for identifying patients at elevated risk of splanchnic vascular complications.

Our findings have several clinical implications. Early risk stratification remains a central goal of acute pancreatitis management, as timely recognition of patients at risk for severe disease permits appropriate monitoring, intensive supportive therapy, ICU admission when indicated, and prompt transfer to tertiary referral centers. Although validated scoring systems such as APACHE II, BISAP, and MCTSI remain valuable, serial inflammatory parameters could complement these tools for patient monitoring. Our data suggest that a CAR-T24 value above 5.31, the Youden-optimal threshold corresponding to a sensitivity of 81.8% and specificity of 78.3% for SAP, may provide prognostic information beyond the admission value alone. Rather than replacing established prognostic scores, serial CAR assessment should be viewed as a readily available complementary tool. An elevated CAR-T24 may identify patients who warrant closer surveillance or escalation of care despite apparently reassuring initial clinical findings, whereas persistently low values may support a lower-intensity monitoring approach in patients with an uncomplicated early course. Conversely, the poor discriminative performance of admission NLR in this cohort suggests that NLR alone may be insufficient for early risk stratification in AP. Future studies should prospectively validate the 24 h CAR threshold identified here, evaluate its performance across different AP etiologies and healthcare settings, and assess whether its integration into established scoring models such as BISAP or APACHE II further improves their discriminatory performance and clinical utility.

The strengths of the present study deserve consideration. Unlike most previous investigations evaluating only admission CAR, the present study assessed serial measurements at admission, 24 h, and 48 h, enabling evaluation of the dynamic inflammatory trajectory during the critical early phase of the disease. CRP and albumin were available for all 150 patients at all three time points, eliminating missing-data bias from the serial analyses. Consecutive patient enrollment was applied, partially mitigating the selection bias inherent in retrospective designs. The study directly evaluated the incremental prognostic contribution of serial CAR beyond admission measurement using multivariable regression, demonstrating the independent predictive value of CAR-T24 after adjustment for age and BUN, rather than merely confirming the known association between baseline CAR and disease severity. Finally, all outcomes were defined using the 2012 Revised Atlanta Classification, ensuring standardized and reproducible severity assessment.

A preliminary analysis of 40 patients, representing 26.7% of the 150-patient cohort included in the present study, was previously published by our group [28]. These 40 patients were admitted between January and August 2024 and constitute a subset of the current cohort. The previous study was explicitly designed and reported as a preliminary analysis and evaluated CAR only at the initial assessment, without serial measurements. The present study substantially extends that preliminary work by increasing the cohort from 40 to 150 patients and, more importantly, by addressing a different primary research question: whether reassessment of CAR during the first 48 h provides incremental prognostic information beyond the admission measurement. Accordingly, the present analyses include CAR at admission, 24 h, and 48 h; changes in CAR over time (ΔCAR); multivariable models evaluating the incremental contribution of serial CAR beyond admission CAR; formal comparison of discriminative performance using DeLong’s method; comparison with admission NLR; and exploratory analyses of selected complications. Thus, while the present cohort partially overlaps with the previously published preliminary cohort, the principal analyses and research question reported here have not been previously published.

Several limitations must be acknowledged. The retrospective single-center design introduces selection bias and limits generalizability to other populations and healthcare settings. The sample size of 150 patients, including 44 SAP events, while consistent with many published biomarker studies, remains relatively limited and provides reduced statistical power for secondary outcomes. In particular, only 10 patients developed splanchnic venous thrombosis, resulting in limited statistical precision. Therefore, the observed association between CAR-T24 and thrombotic complications should be considered preliminary and hypothesis-generating and requires confirmation in larger cohorts specifically powered for this outcome. The timing of symptom onset prior to hospital admission varied between patients and could not be standardized, potentially introducing variability into biomarker values at all three time points and affecting both absolute CAR and ΔCAR. In our analysis, NLR was assessed only at admission, whereas CAR was evaluated serially, making a direct comparison of their prognostic performance methodologically asymmetric. Although the prespecified multivariable models underwent internal validation using 2000 bootstrap resamples with optimism correction, no independent cohort was available for external validation. External validation was beyond the scope of the present study, whose primary aim was to investigate the incremental prognostic contribution of serial CAR reassessment rather than to develop a definitive clinical prediction tool. Accordingly, the models should be regarded as internally validated exploratory prognostic models, and their transportability, calibration, and clinical utility require confirmation in independent, preferably prospective multicenter cohorts across different acute pancreatitis etiologies and healthcare settings before routine clinical implementation. Another limitation is the absence of a direct head-to-head comparison between CAR-T24 and established multidimensional bedside severity scores such as BISAP or APACHE II. Consequently, the present findings demonstrate the incremental prognostic contribution of CAR-T24 beyond the prespecified variables age, BUN, and admission CAR, but do not establish superiority over currently available clinical scoring systems. Future prospective studies should directly compare these approaches and determine whether incorporating serial CAR into established severity scores provides additional clinically meaningful discrimination, calibration, or decision utility.

The optimal timing of CAR reassessment warrants systematic evaluation. Integration of serial CAR into composite prediction models combining laboratory, clinical, and radiological variables (such as BISAP, APACHE II, or the Ranson score) may further improve early risk stratification and should be evaluated in dedicated model-development studies. Prospective studies should also include serial NLR measurements alongside serial CAR, enabling a symmetric comparative assessment that the present retrospective design could not provide. The potential relationship between persistent systemic inflammation, elevated CAR, and thrombo-inflammatory mechanisms contributing to splanchnic venous thrombosis deserves dedicated prospective investigation in larger cohorts with sufficient thrombotic event rates. Ultimately, whether a serial CAR-guided triage strategy translates into improved clinical outcomes, through timely escalation of care or earlier transfer to tertiary centers, deserves future research.

## 5. Conclusions

In conclusion, serial assessment of the C-reactive protein-to-albumin ratio represents a simple, inexpensive, and readily implementable approach to early risk stratification in acute pancreatitis. Among the time points evaluated, CAR-T24 demonstrated the strongest prognostic performance and, when added to a base model comprising age, BUN, and admission CAR, yielded a statistically significant improvement in discriminative accuracy (AUC 0.890 vs. 0.841). Dynamic changes in CAR over time (ΔCAR) were not independently associated with severe disease, suggesting that the absolute inflammatory burden at a standardized reassessment time point is more informative than the magnitude of change from baseline. The 24 h model retained good discrimination and calibration after bootstrap internal validation; however, external validation in independent cohorts is required before clinical implementation.

Admission NLR demonstrated poor discriminative performance in this cohort (AUC 0.578) and did not provide additional prognostic value over serial CAR. An exploratory association between elevated serial CAR and splanchnic venous thrombosis was also identified, warranting prospective investigation in larger cohorts. Serial CAR assessment may serve as a practical complement to established clinical scoring systems for identifying patients at risk of severe acute pancreatitis and its complications.

## Figures and Tables

**Table 1:** Comparison of demographic, clinical and laboratory parameters between severity groups.

Variable	Total (*n* = 150)	Non-Severe AP (*n* = 106)	Severe AP (*n* = 44)	*p*-Value
Age, years	55.00 (43.00–69.00)	53.00 (40.00–69.00)	57.00 (48.25–70.00)	0.176
Male sex, *n* (%)	85 (56.7%)	53 (50.0%)	32 (72.7%)	0.017
Biliary etiology, *n* (%)	84 (56.0%)	61 (57.5%)	23 (52.3%)	0.680
Alcohol-related etiology, *n* (%)	56 (37.3%)	37 (34.9%)	19 (43.2%)	0.442
Pancreatic pseudocyst, *n* (%)	12 (8%)	6 (5.7%)	6 (13.6%)	0.111
Splanchnic venous thrombosis, *n* (%)	10 (6.7%)	2 (1.9%)	8 (18.2%)	0.001
Pancreatic ascites, *n* (%)	45 (30%)	30 (28.3%)	15 (34.1%)	0.558
BUN (mg/dL)	13.08 (8.99–17.16)	13.08 (8.06–16.35)	13.54 (9.81–19.50)	0.080
Creatinine, mg/dL	0.73 (0.63–0.90)	0.70 (0.63–0.82)	0.79 (0.67–0.99)	0.097
NLR	5.16 (2.52–9.57)	4.59 (2.44–7.93)	6.13 (3.26–10.00)	0.131
CRP at admission, mg/L	145.00 (82.25–212.50)	126.50 (62.75–167.00)	228.50 (159.50–271.00)	<0.001
CRP at 24 h, mg/L	134.00 (76.00–187.00)	120.00 (65.00–153.50)	210.00 (172.25–258.75)	<0.001
CRP at 48 h, mg/L	62.00 (27.00–102.75)	40.50 (23.00–75.00)	123.00 (82.00–156.00)	<0.001
Albumin at admission, g/L	35.0 (33.0–37.0)	36.0 (34.0–37.0)	33.5 (31.0–35.0)	<0.001
Albumin at 24 h, g/L	33.0 (30.0–35.0)	34.0 (31.2–36.0)	29.0 (28.0–32.0)	<0.001
Albumin at 48 h, g/L	31.0 (28.0–33.8)	32.0 (30.0–35.0)	27.0 (25.0–31.0)	<0.001
CAR-T0	4.12 (2.47–6.40)	3.48 (1.61–4.60)	6.87 (4.63–8.66)	<0.001
CAR-T24	4.25 (2.25–6.23)	3.33 (1.81–4.64)	7.32 (5.31–8.81)	<0.001
CAR-T48	1.90 (0.81–3.61)	1.25 (0.68–2.39)	4.28 (2.92–5.75)	<0.001
MCTSI	5.00 (4.00–7.75)	4.00 (4.00–5.00)	8.00 (8.00–9.00)	<0.001
ICU admission, *n* (%)	20 (13.3%)	0 (0.0%)	20 (45.5%)	<0.001
In-hospital mortality, *n* (%)	3 (2%)	0 (0.0%)	3 (6.8%)	0.002
Paralytic ileus, *n* (%)	51 (34.0%)	33 (31.1%)	18 (40.9%)	0.336
Duration of hospitalization, days	6 (5.00–8.00)	6 (5.00–7.00)	8 (6.75–10.00)	<0.001

Abbreviations: AP, acute pancreatitis; BUN, blood urea nitrogen; CAR, C-reactive protein/albumin ratio; CRP, C-reactive protein; MCTSI, Modified Computed Tomography Severity Index; NLR, neutrophil-to-lymphocyte ratio; ICU, intensive care unit.

**Table 2:** Univariable association between CAR and severe acute pancreatitis.

Predictor	OR	95% CI	*p*-Value	AUC	Cutoff	Sensitivity (%)	Specificity (%)
CAR-T0	1.663	1.384–1.998	<0.001	0.829 (0.761–0.898)	4.63	79.5	73.6
CAR-T24	1.926	1.554–2.386	<0.001	0.874 (0.816–0.932)	5.31	81.8	78.3
CAR-T48	2.121	1.645–2.735	<0.001	0.857 (0.791–0.922)	2.92	77.3	80.2
ΔCAR-T24	1.196	0.937–1.526	0.151	0.592	-	-	-
ΔCAR-T48	0.838	0.683–1.027	0.089	0.599	-	-	-

Abbreviations: AUC, area under the receiver operating characteristic curve; CAR, C-reactive protein/albumin ratio; CI, confidence interval; ΔCAR-T24, change in CAR from admission to 24 h; ΔCAR-T48, change in CAR from admission to 48 h; OR, odds ratio.

**Table 3:** Multivariable logistic regression models for severe acute pancreatitis using admission and serial CAR measurements.

Model	Age, OR (95% CI); *p*	BUN, OR (95% CI); *p*	CAR-T0, OR (95% CI); *p*	CAR-T24, OR (95% CI); *p*	CAR-T48, OR (95% CI); *p*	Model AUC	Improvement vs. Base Model
Base model: Age + BUN + CAR-T0	1.007 (0.978–1.036); 0.636	1.050 (1.002–1.101); 0.040	1.650 (1.363–1.997); <0.001	—	—	0.841	—
Base model + CAR-T24	1.013 (0.981–1.045); 0.438	1.056 (1.000–1.114); 0.050	1.025 (0.773–1.360); 0.864	1.916 (1.387–2.647); <0.001	—	0.890	*p* < 0.001
Base model + CAR-T48	1.005 (0.975–1.037); 0.736	1.047 (0.993–1.104); 0.090	1.257 (0.971–1.627); 0.083	—	1.647 (1.177–2.305); 0.004	0.868	*p* = 0.002

Abbreviations: AUC, area under the receiver operating characteristic curve; BUN, blood urea nitrogen; CAR, C-reactive protein/albumin ratio; CI, confidence interval; OR, odds ratio.

**Table 4:** Exploratory associations of serial CAR with local complications.

Outcome	Predictor	OR (95% CI)	*p*-Value	AUC
Pseudocyst	CAR-T24	1.141 (0.928–1.404)	0.211	0.587
Pseudocyst	CAR-T48	1.147 (0.892–1.475)	0.284	0.585
Splanchnic venous thrombosis	CAR-T24	1.691 (1.275–2.243)	<0.001	0.825
Splanchnic venous thrombosis	CAR-T48	1.645 (1.244–2.176)	<0.001	0.819
Pancreatic ascites	CAR-T24	1.021 (0.897–1.162)	0.751	0.505
Pancreatic ascites	CAR-T48	1.076 (0.914–1.267)	0.379	0.519

Abbreviations: AUC, area under the receiver operating characteristic curve; CAR, C-reactive protein/albumin ratio; CI, confidence interval; OR, odds ratio.

**Table 5:** Pairwise comparisons of the discriminative performance of CAR and NLR for predicting severe acute pancreatitis.

Comparison	AUC 1	AUC 2	ΔAUC	95% CI for ΔAUC	DeLong *p* Value
CAR-T24 vs. CAR-T0	0.874	0.829	0.045	−0.003 to 0.093	0.067
CAR-T24 vs. NLR	0.874	0.578	0.296	0.173 to 0.419	<0.001
CAR-T0 vs. NLR	0.829	0.578	0.251	0.127 to 0.375	<0.001
CAR-T48 vs. CAR-T0	0.857	0.829	0.027	−0.029 to 0.084	0.342
CAR-T48 vs. NLR	0.857	0.578	0.278	0.160 to 0.397	<0.001

Abbreviations: AUC, area under the receiver operating characteristic curve; CI, confidence interval; ΔAUC, absolute difference between the two compared AUCs. *p* values were obtained using DeLong’s test for correlated ROC curves.

**Table 6:** Internal bootstrap validation of the prespecified multivariable models for severe acute pancreatitis.

Model	Apparent AUC (95% CI)	Mean AUC Optimism	Optimism-Corrected AUC	Apparent Brier Score	Optimism-Corrected Brier Score	Corrected Calibration Intercept/Slope
Admission: age + BUN + CAR-T0	0.841 (0.768 to 0.903)	0.009	0.831	0.139	0.147	0.005/0.953
Primary 24 h: age + BUN + CAR-T0 + CAR-T24	0.890 (0.826 to 0.943)	0.012	0.877	0.116	0.127	0.013/0.919
Secondary 48 h: age + BUN + CAR-T0 + CAR-T48	0.868 (0.800 to 0.928)	0.015	0.853	0.126	0.136	0.008/0.920

## Data Availability

The data supporting the findings of this study are available from the corresponding author upon reasonable request. The data are not publicly available due to privacy and ethical restrictions related to patient confidentiality.

## Supplementary Materials

The following supporting information can be downloaded at: https://www.mdpi.com/article/10.3390/life16091564/s1, Table S1: Coefficients and equations for the locked logistic regression models.

## Abbreviations

The following abbreviations are used in this manuscript:

Abbreviation	Definition
ALT	Alanine aminotransferase
AP	Acute pancreatitis
APACHE II	Acute Physiology and Chronic Health Evaluation II
AST	Aspartate aminotransferase
AUC	Area under the receiver operating characteristic curve
BISAP	Bedside Index for Severity in Acute Pancreatitis
BUN	Blood urea nitrogen
CAR	C-reactive protein-to-albumin ratio
CAR-T0	C-reactive protein-to-albumin ratio at admission
CAR-T24	C-reactive protein-to-albumin ratio 24 h after admission
CAR-T48	C-reactive protein-to-albumin ratio 48 h after admission
CECT	Contrast-enhanced computed tomography
CI	Confidence interval
CRP	C-reactive protein
CT	Computed tomography
DAMPs	Damage-associated molecular patterns
ΔAUC	Difference in area under the receiver operating characteristic curve
ΔCAR-T24	Change in CAR from admission to 24 h
ΔCAR-T48	Change in CAR from admission to 48 h
ICU	Intensive care unit
IL-1β	Interleukin-1 beta
IL-6	Interleukin-6
IQR	Interquartile range
MCTSI	Modified Computed Tomography Severity Index
NLR	Neutrophil-to-lymphocyte ratio
NLR-T0	Admission neutrophil-to-lymphocyte ratio
OR	Odds ratio
ROC	Receiver operating characteristic
SAP	Severe acute pancreatitis
SIRS	Systemic inflammatory response syndrome
TNF-α	Tumor necrosis factor alpha
VIF	Variance inflation factor
