# Supplementary material for: Dynamic Assessment of the C-Reactive Protein-to-Albumin Ratio Improves Early Prediction of Severe Acute Pancreatitis: A Retrospective Cohort Study
Source: Life (Basel). 2026 Sep 17;16(9):1564. doi: 10.3390/life16091564 (PMC13608710; doi:10.3390/life16091564)
Supplement: Supplementary file 1 [file life-16-01564-s001.zip › life-4531846-supplementary.pdf]

Supplementary material

Supplementary Table S1. Coefficients and equations for the locked logistic regression models

Model	Intercept	Age beta	BUN beta	CAR-T0 beta	CAR-T24 beta	CAR-T48 beta
Admission	-4.570836	0.006951	0.049049	0.500645	—	—
Primary 24-hour	-5.959753	0.012491	0.053994	0.024732	0.650240	—
Secondary 48-hour	-4.467177	0.005322	0.045777	0.228599	—	0.498928
